# Predictive value of the American college of surgeons “surgical risk calculator” (ACS-NSQIP SRC) for plastic and reconstructive surgery: a validation study from an academic tertiary referral center in Germany

**DOI:** 10.1186/s13037-025-00438-y

**Published:** 2025-04-30

**Authors:** Florian Bucher, Martynas Tamulevicius, Nadjib Dastagir, Catherine Fuentes Alvarado, Doha Obed, Khaled Dastagir, Peter M. Vogt

**Affiliations:** Department of Plastic, Aesthetic, Hand and Reconstructive Surgery, Hannover Medical School, Carl-Neuberg-Strasse 1, 30625 Hannover, Germany

**Keywords:** Risk calculator, ACS-NSQIP, Body contouring, Breast reconstruction

## Abstract

**Aims:**

The American College of Surgeons Surgical Risk Calculator (ACS-NSQIP SRC) was designed to predict morbidity and mortality in order to help providing informed consent. This study evaluated its performance in the field of plastic and reconstructive surgery for patients undergoing body contouring and breast reconstruction procedures.

**Methods:**

A retrospective analysis of patients undergoing body contouring and breast reconstruction procedures from January 1, 2022 to November 1, 2024 was performed.

**Results:**

The ACS-NSQIP SRC showed good prediction only for severe complications in patients undergoing breast reconstruction with DIEP flap (AUC = 0.727); overall prediction and calibration for the remaining 15 subgroups was poor. The incidence of overall and general complications, as well as length of hospital stay was underestimated.

**Conclusions:**

The overall performance of the ACS-NSQIP SRC was poor, a finding that underlines the importance of individual decision-making, also considering the surgeon’s expertise and patient-specific characteristics.

## Introduction

**Fig. 1:**
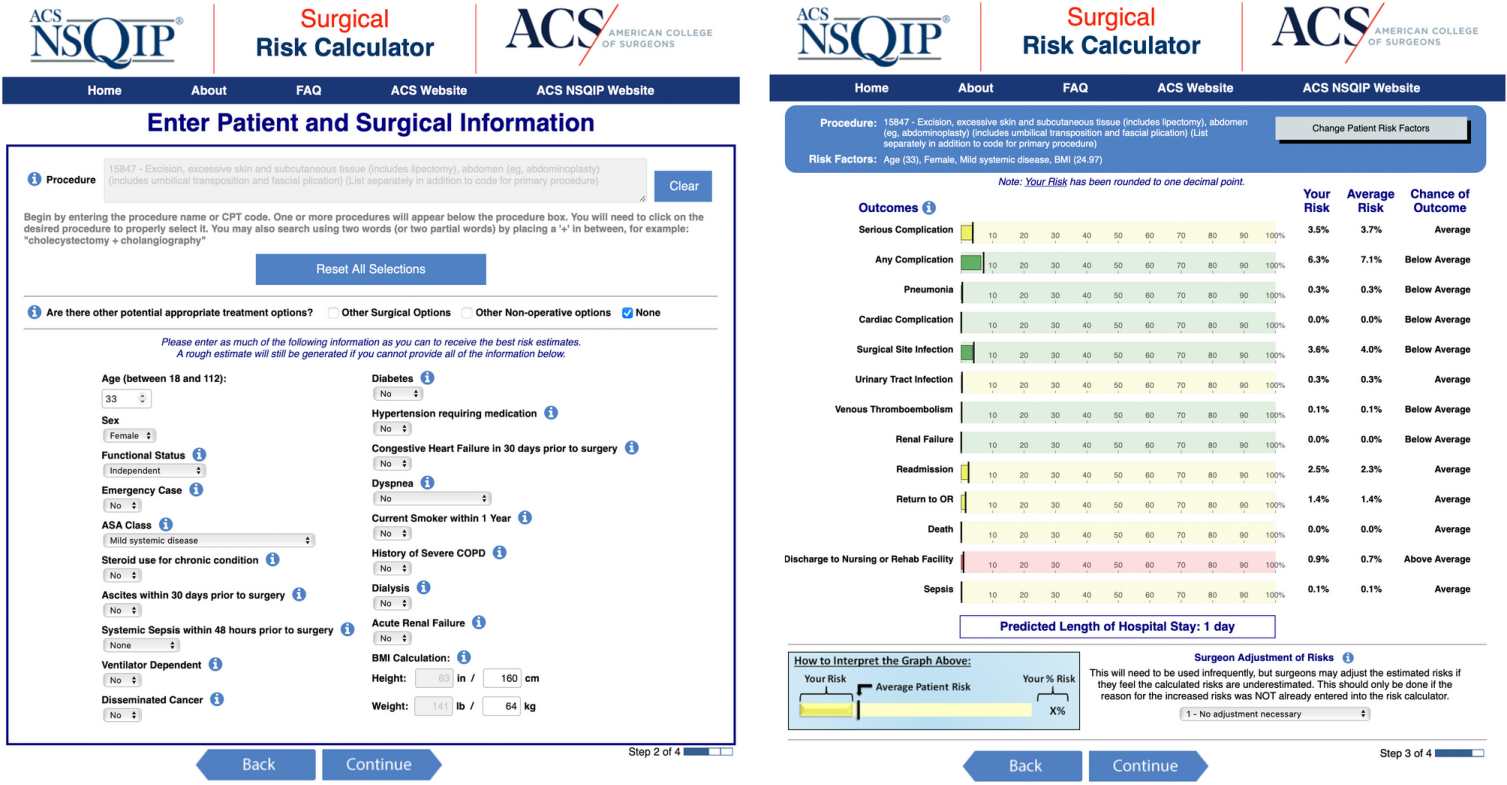
Exemplary input and risk prediction of the ACS risk calculator

Body contouring surgery is a growing area in plastic and reconstructive surgery that may be required after massive weight loss, in case of breast hypertrophy or to enhance aesthetic appearance. Over 1.1 million abdominoplasties, 680,000 breast reductions and 70,000 upper body lifts have been performed worldwide [1]. Before obtaining informed consent, the patient has to be educated about general and specific complications and their relative probability [2]. Besides providing accurate preoperative risk assessment for a patient to facilitate their informed decision-making, the national health system often emphasizes surgical risk as a quality benchmark [3]. Over the past years, multiple surgical risk calculators (SRCs) have been proposed [4–6]. They aim to predict postoperative outcomes based on several individual risk factors of patients. The American College of Surgeons (ACS) developed a risk calculator based on clinical data from 1.4 million cases in 393 hospitals that participate in the National Quality Surgical Improvement Program (NSQIP) between 2009 and 2012 [7]. The tool may be accessed by patients and health care providers online free of charge. The planned procedure is entered via current procedural terminology (CPT) and 19 patient-related preoperative risk factors are required. Based on these variables, overall complications, serious complications, and length of stay are predicted. The chances of outcome are presented in percentages and interpreted relative to the overall cohort (below average, average and above average). The predicted risk may be adjusted by applying a “surgeon adjustment of risk”. Figure 1 shows the input page of the American College of Surgeons surgical risk calculator (ACS-NSQIP SRC) and the provided outcome. Previous studies that aimed to validate ACS-NSQIP SRC for international patients or specific procedures reported mixed performance for the predicted outcomes [8–10]. The literature is currently lacking a validation of ACS-NSQIP SRC for plastic and reconstructive surgeries.

Hence, the aim of the study was to evaluate the performance and thus the applicability of the ACS-NSQIP SRC for German patients from a high-volume university hospital in Northern Germany regarding body contouring and breast reconstruction procedures.

## Methods

### Study design

A retrospective analysis of patients undergoing abdominoplasty (CPT code 15847), breast reduction surgery (CPT code 19318), and breast reconstruction with free deep Inferior Epigastric Perforator flap (DIEP) (CPT code 19364) or latissimus dorsi flap (CPT code 19361) from January 1, 2022 to November 1, 2024 was performed. Routine data regarding preoperative risk factors were extracted anonymously from the department’s internal registry respecting the national data protection protocols. The patients or their legal representative signed an informed consent form to allow anonymous data analysis. Given the retrospective analysis of an anonymous database, approval by the local ethics committee was waived.

The exclusion criteria included patients under 18 years of age, missing data regarding the required preoperative risks for the ACS-NSQIP SRC, and missing consent for data use.

For each patient the ACS-NSQIP SRC (https://riskcalculator.facs.org/) was used to calculate the patient-specific outcome. The study design takes into account the Strengthening the Reporting of Observational Studies in Epidemiology (STROBE)-guidelines.

### Statistical analysis

Statistical analyses were conducted using GraphPad Prism 10.3.1 (GraphPad Software, Inc., La Jolla, CA, USA), Microsoft Excel 16.78 (Microsoft Corp., Redmon, WA, USA), IBM SPSS 30 (IBM Corp., Armonk, NY, USA), and Numbers 12.2.1 (Apple Inc., Cupertino, CA, USA). Descriptive statistics are presented as numbers (percentage) and medians (interquartile range). The predicted outcome was grouped into a positive occurrence (positive) and a non-occurrence (negative) and analyzed separately using the Mann-Whitney U test.

The C-statistic (equivalent to the area under the curve) was calculated; representing the incidence in the outcome of the patient cohort compared with the predicted outcome probability calculated by the ACS-NSQIP SRC through a logistic regression model. A C-statistic close to 0.5 indicates random concordance, while a C-statistic close to 1.0 represents a perfect prediction. A value above 0.7 is considered acceptable [11]. Visual representation was done using receiver operating characteristic (ROC) curves.

The Brier score is calculated as the squared mean of the difference between the outcome and the predicted probability of the ACS-NSQIP SRC. A Brier score close to 0 indicates a high predictive accuracy and is considered a marker of calibration [12].

A boxplot highlighting the length of hospital stay was generated and the variables were analyzed using a one-sample t-test.

## Results

### Patient demographics and characteristics

A total of 90 patients with a mean age of 41 years (18–69 years, SD ± 13.2 years) were included in this study. Of those, 30 patients underwent abdominoplasties, while 30 patients received bilateral breast reduction surgery. Regarding breast reconstructions, 30 patients opted for free microvascular DIEP-flap reconstruction and 10 patients received breast reconstruction with a latissimus dorsi flap. The mean body mass index (BMI) was 26.8 kg/m^2^ (SD (± 4.6 kg/m^2^) and the male-to-female ratio was 4:41. All procedures were elective, and the majority of patients was classified as American Society of Anesthesiologists (ASA) class II. The most common comorbidity was arterial hypertension (18.9%), followed by diabetes mellitus requiring oral medication. Twelve (13.3%) patients were active smokers up to 1 year prior to surgery. The Patient characteristics and comorbidities are presented in Table 1.

**Table 1:**
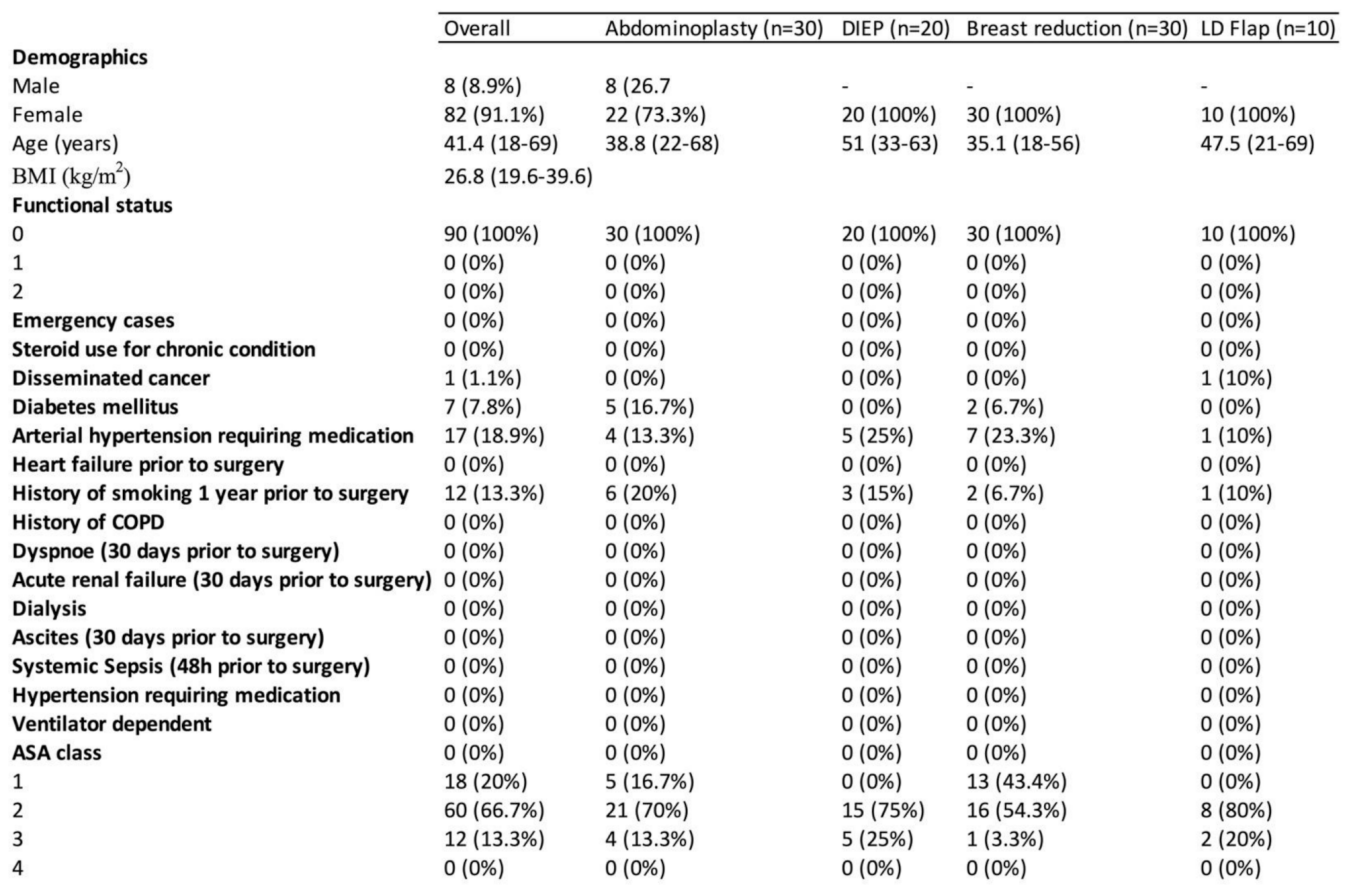
Patient demographics and preoperative risk factors

### Patient characteristics and short-term outcomes

**Fig. 2:**
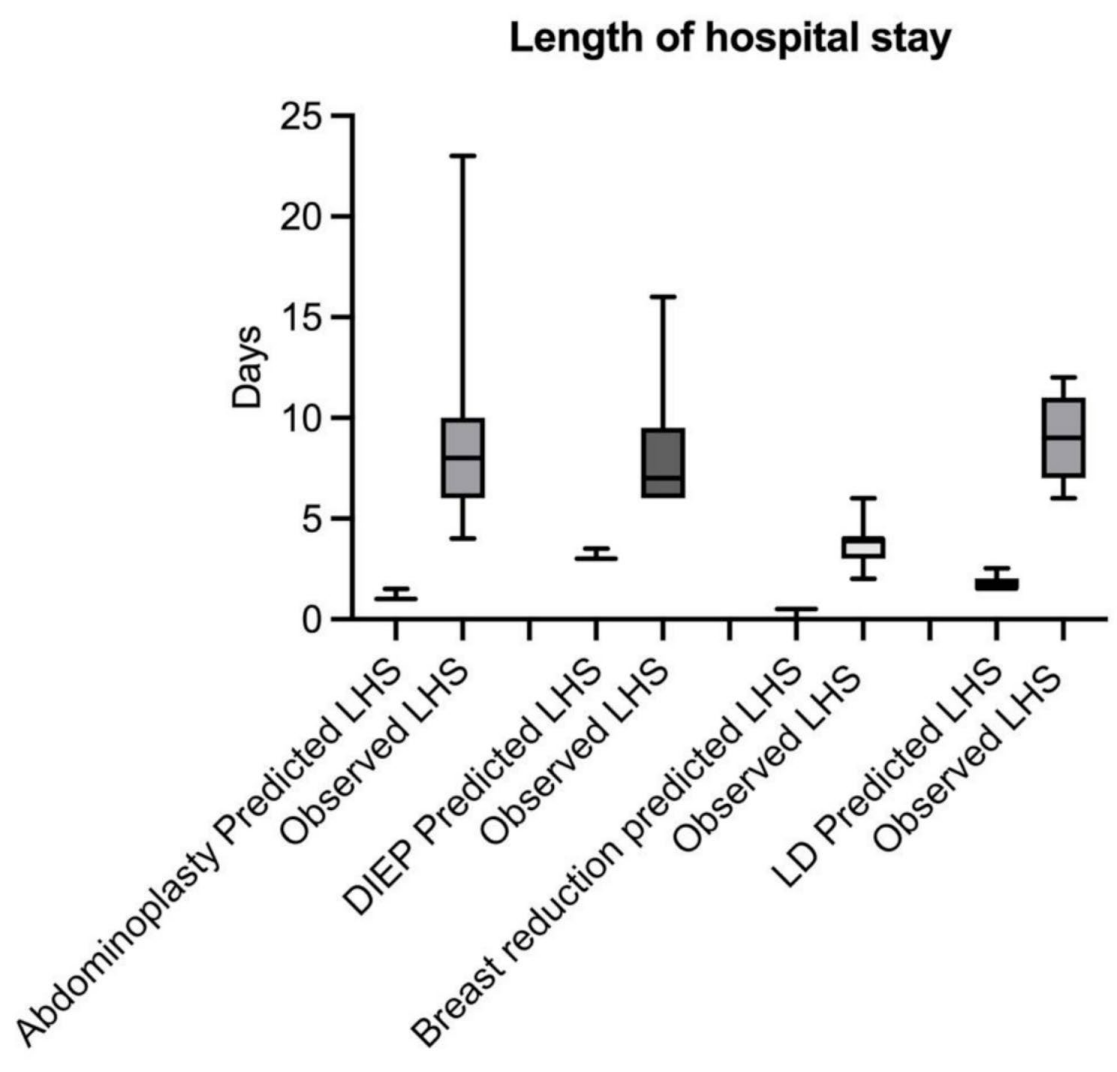
Boxplot comparing observed and predicted length of hospital stay

A total of 16 patients (17.8%) suffered complications and 11 patients (12.2%) experienced severe complications which led to surgical revision in 9 cases (10%). The most common complication was a surgical site infection in 9 cases (10%). No patients experienced renal failure, pneumonia, cardiac complications, venous thromboembolism, or sepsis. The overall length of hospital stay was 7 days, which was significantly longer compared with the prediction of 1.4 days (*p* < 0.0001). Figure 2 shows a boxplot of the observed and predicted length of hospital stay for each subgroup. No patients were discharged to a nursing or rehab facility.

### Predicted and observed outcome

**Fig. 3:**
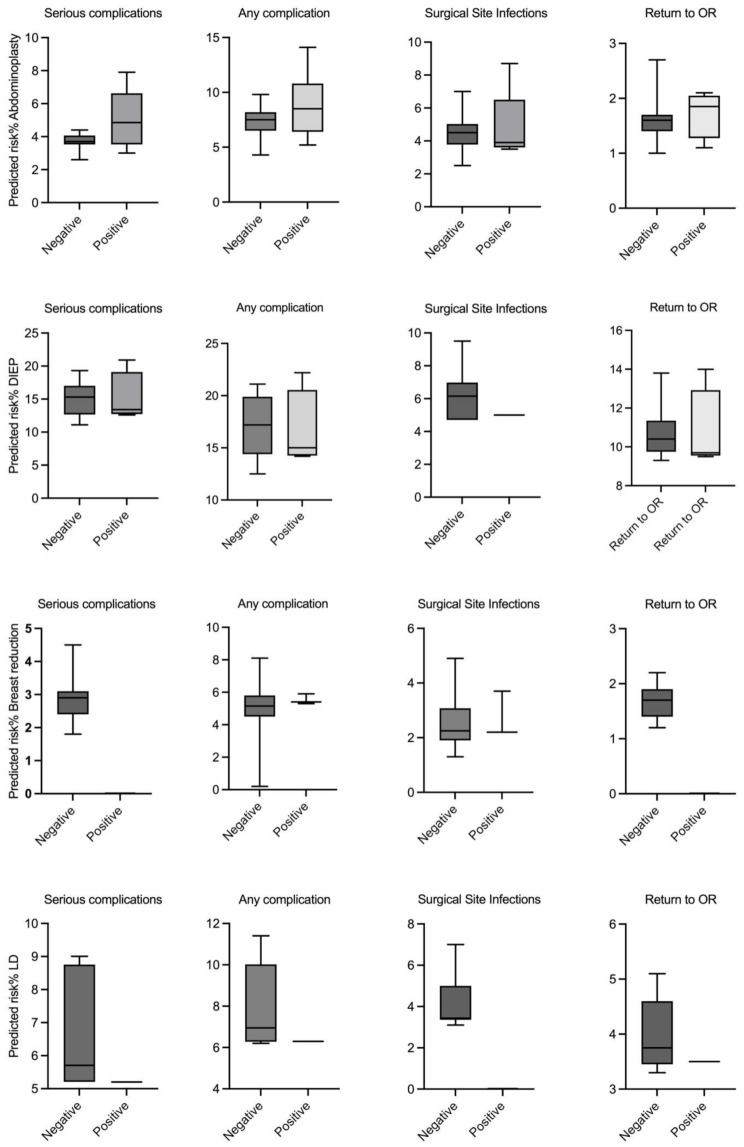
Predicted risk stratified by observed outcome

To compare the predicted risk, the patients in each subgroup were divided according to the occurrence of each complication (positive) and non-occurrence (negative). The predicted risks are summarized in Fig. 3. There was no statistically significant correlation between the predicted risk of individual complications among the subgroups.

Table 2 summarizes the C-statistic and Brier scores for the predicted risk by the ACS-NSQIP SRC. Discrimination of the ACS-NSQIP SRC was poor for almost all outcomes, denoted by a C-statistic < 0.7. The only prediction providing acceptable results with an AUC = 0.727 was for severe complications in patients who underwent breast reconstruction with a DIEP-flap.

**Table 2:**
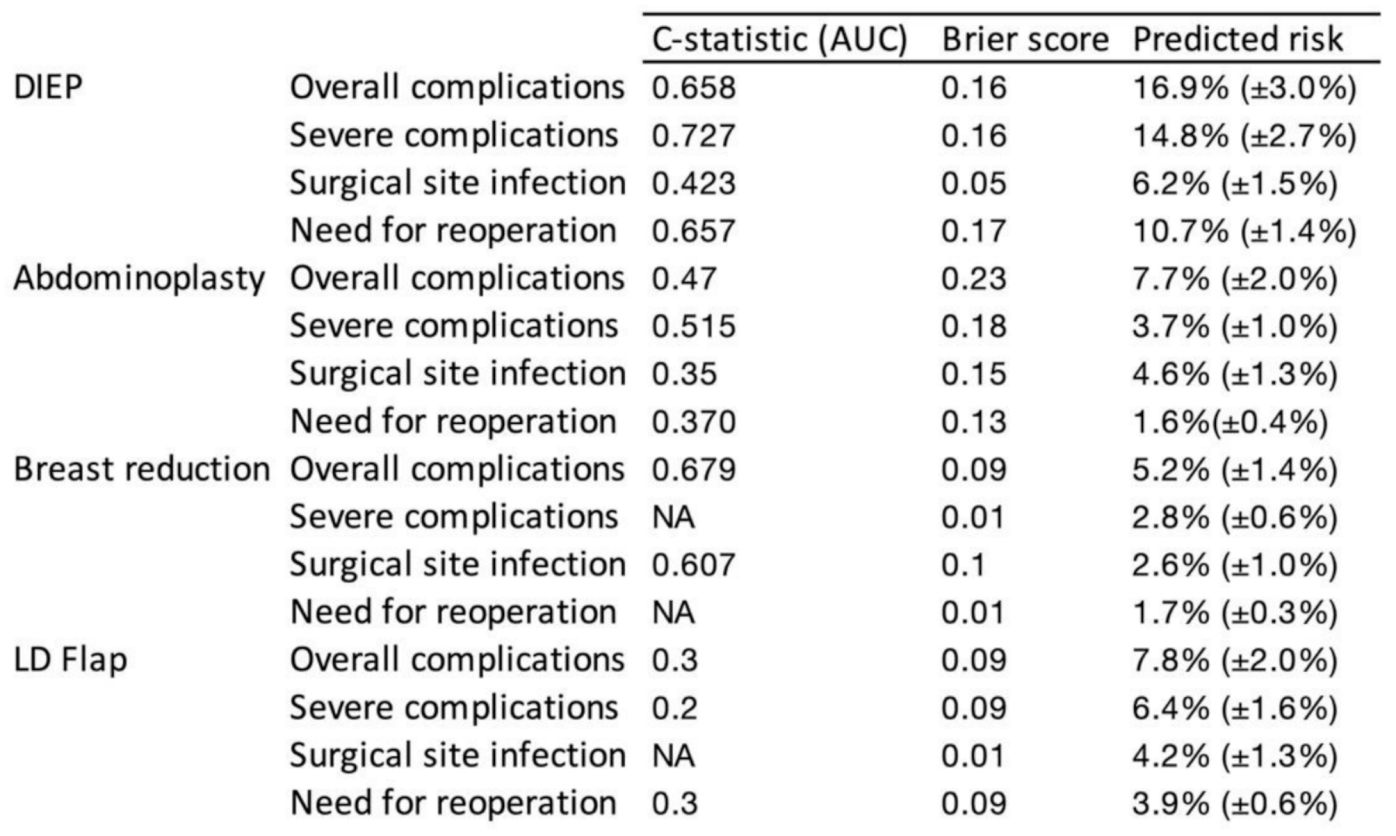
C-statistics (AUC) and Brier score for complications and need for reoperation

**Fig. 4:**
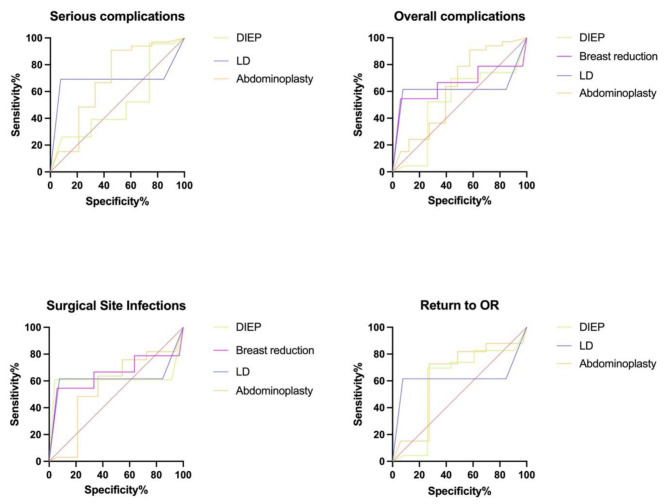
C-statistic ROC curves for complications

Calibration was good for patients undergoing breast reduction surgery and breast reconstruction using latissimus dorsi flap (Brier score ≤ 0.1). ROC curves as visual representation of C-statistic is shown in Fig. 4. Prediction of the ACS-NSQIP SRC for each subgroup showed poor prediction for every considered outcome, as the graph fell below the random classification line for subgroup.

## Discussion

Adequate surgical risk estimation has remained a challenge for surgeons for decades. Perioperative risk estimation is multifactorial, and includes patient-specific comorbidities, functional capacity estimation, and laboratory values [13]. The outcome may be influenced by multiple parameters, such as an accurate indication, hospital volume, and the surgeon’s experience [14, 15]. A risk calculator may serve as a comprehensive assessment tool that provides both patients and health care providers with the expected postoperative outcome and thus gives the patient valuable insight on the expected morbidity and mortality [16]. With the rising impact of artificial intelligence (AI), there is a question whether the surgeon’s experience is superior in predicting complications compared with risk calculator or AI models. The ACS-NSQIP SRC was created to provide easily understandable information regarding the postoperative complication rates after specific surgical procedures. Previous studies have focused on procedure-specific evaluations with no regard to the overall specialty levels [17].

This is the first study that evaluated the performance of the ACS-NSQIP SRC in the field of plastic and aesthetic surgery, and this approach has provided valuable insight into its applicability.

The ACS-NSQIP SRC provides the outcome for plastic surgery procedures such as abdominoplasties, breast reduction surgery and breast reconstruction while, but other procedures such as liposuction and burn care are not included. This study focused on body contouring surgery and breast reconstruction. The overall prediction and calibration of the ACS-NSQIP SRC was poor for 15 out of 16 subgroups. The only good prediction was shown for severe complications in patients undergoing breast reconstruction with DIEP flap (AUC = 0.727). Overall calibration was poor with a maximum Brier score 0.23. The performance of the ACS-NSQIP SRC in previously published studies remains debatable. Poor performance with underestimation of surgical risk has been described for complex general surgery procedures such as hepato-pancreato-biliary surgery [9, 18]. A poor predictive ability was also concluded by Hamade et al. for hysterectomies [19] and various gynecologic oncology procedures [20]. Similar findings of poor prediction power of ACS-NSQIP SRC were obtained for oncologic urology. Authors attributed these findings to inadequate consideration of nutritional status and procedure complexity [21, 22]. In their meta-analysis, Goodwin et al. reported an overall good prediction of complications for acute care, colorectal; orthopedic surgery, ENT; and cardiothoracic surgery. However, there was an overall underappreciation of surgical risk of up to 9.8% [17].

The ACS-NSQIP SRC has several limitations. It is based on data from hospitals participating in the NSQIP. However, this accounts for only approximately 10% of U.S. hospitals which perform approximately 30% of surgeries [7]. Consequently, this leads to underrepresentation of data. Furthermore, preconditions, surgical techniques and recovery treatment have changed in all surgical specialties. Yet the ACS-NSQIP SRC uses data collected approximately 20 years ago and may be considered outdated. Lastly, the ACS-NSQIP SRC includes more than 1,500 CPT codes across all surgical subspecialities. The American Medical Association changes this catalogue annually by adding, deleting or revising CPT codes. The latest CPT release contains 420 updates which are not represented ACS-NSQIP SRC [23].

Regarding the field of plastic and reconstructive surgery, several limitations of the ACS-NSQIP SRC should be considered. Risk cannot be predicted for the combination of procedures such as body contouring surgery and liposuction, because the ACS-NSQIP SRC does not allow multiple CPT codes. As mentioned above, the nutritional status is not considered. This may be important for patients who had undergone surgical weight-loss procedures and have now opted for body contouring procedures following massive weight loss [24].

These patients may also have diabetes mellitus which is considered a major risk factor for delayed wound healing. The ACS-NSQIP SRC does consider the impact of good glycemic control measured by glycated hemoglobin [25]. Furthermore, the ACS-NSQIP SRC does not consider previous radiation therapy or chemotherapy, which may adversely affect overall outcome and wound healing.

This study has several limitations. First this study was retrospective: The ACS-NSQIP SRC was used for retrospective risk calculation and not in the pre-operative setting. Second, the monocentric study cohort was relatively small. Lastly, this cohort comprised only a large area of northern Germany while the ACS-NSQIP SRC comprises nationwide data across the U.S.

In the future pooled, multicentric data would be required to provide more insight on the prediction capability of the ACS-NSQIP SRC.

Overall, surgical risk calculators may provide valuable information for both health care personnel and patients. However, the limited generalizability of ACS-NSQIP SRC should be considered. Providing informed consent including surgical risk prediction remains a challenge for every surgeon and is best mastered as symbiosis of surgeon’s experience and individual patient characteristics.

## Conclusion

Overall performance of the ACS-NSQIP SRC in the field of plastic and reconstructive surgery was poor which underlines the importance of individual decision making, considering the surgeon’s expertise and patient-specific characteristics.

## Data Availability

No datasets were generated or analysed during the current study.
